# Improved 3D late gadolinium enhancement MRI for patients with arrhythmia or heart rate variability

**DOI:** 10.1186/1532-429X-15-S1-P29

**Published:** 2013-01-30

**Authors:** Sebastian Weingärtner, Mehmet Akcakaya, Sophie Berg, Kraig V Kissinger, Warren J Manning, Reza Nezafat

**Affiliations:** 1 Department of Medicine, Beth Israel Deaconess Medical Center and Harvard Medical School, Boston, MA, USA; 2 Department of Radiology, Beth Israel Deaconess Medical Center and Harvard Medical School, Boston, MA, USA; 3 Computer Assisted Clinical Medicine, University Medical Center Mannheim, Heidelberg University, Mannheim, Germany

## Background

Arrhythmia and heart rate variability have adverse effects on the image quality of Late Gadolinium Enhancement (LGE). Due to the incomplete recovery after an inversion pulse, changes in the RR-interval length induce a k-space weighting, which results in ghosting artifacts in the images. While the impact of heart-rate variability is less pronounced in 2D LGE due to short scan times (10-15 sec), it often results in non-diagnostic image quality in 3D LGE (scan time 6-10 min). Our aim is to develop a novel LGE imaging sequence for patients with heart rate variability or arrhythmia.

## Methods

In the SAturation Pulse Prepared Heart Rate independent Inversion-REcovery (SAPPHIRE) sequence, a saturation pulse is applied immediately after the ECG R-wave to erases the magnetization history at the beginning of each heartbeat. This saturation pulse is followed by a regular inversion pulse before k-space sampling, analogous to the conventional LGE sequence. To enable nulling of the healthy myocardium, the delays between the saturation and the inversion pulse to the data acquisition are adjusted. For this purpose the longitudinal signal relaxation after the saturation and the inversion pulse is expressed in terms of the Bloch equations and optimal delay times are calculated such that the healthy myocardium is nulled during the data acquisition. Numerical simulations, phantom and in vivo experiments were performed to demonstrate the feasibility of the SAPPHIRE LGE sequence. Based on the Bloch-equations the image acquisition of a numerical phantom with SAPPHIRE LGE and conventional LGE in the presence of arrhythmia was simulated. The numerical phantom parallels a cardiac short axis view with four compartments (LV, RV, myocardium and scar), where the simulated T1-times ranged from 350 ms to 560 ms. Furthermore a bottle phantom (T1 = 360 ms) was imaged with arrhythmic ECG. To simulate arrhythmia in the phantom images and the numerical simulations, multiple ECGs with normal distributed RR-interval lengths were computed, with a mean RR-interval length of 667 ms (i.e., heart rate of 90 bpm) and a standard-deviation of 200 ms - 400 ms (30% - 60% of the mean).

## Results

**Figure 1:**
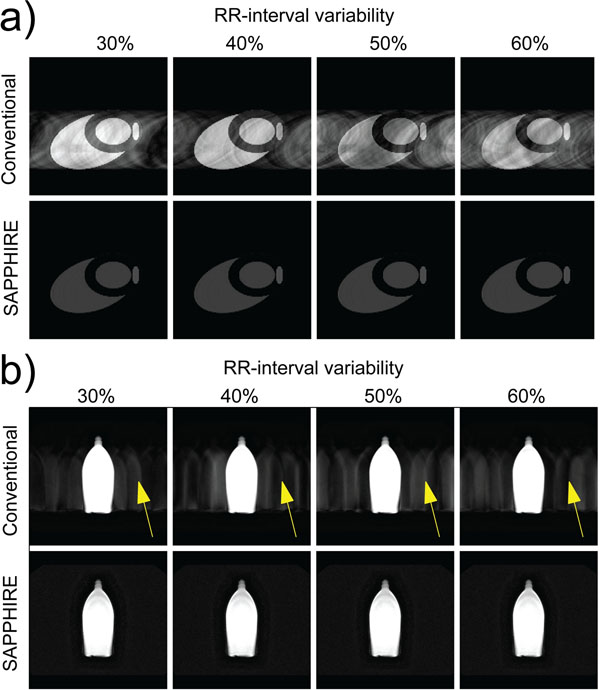
Simulated image acquisition (a) and images of a phantom (b) during arrhythmic ECGs with different RR-interval variability. SAPPHIRE LGE images are free from ghosting artifacts, while conventional LGEs shows ghosting artifacts (yellow arrows).

**Figure 2:**
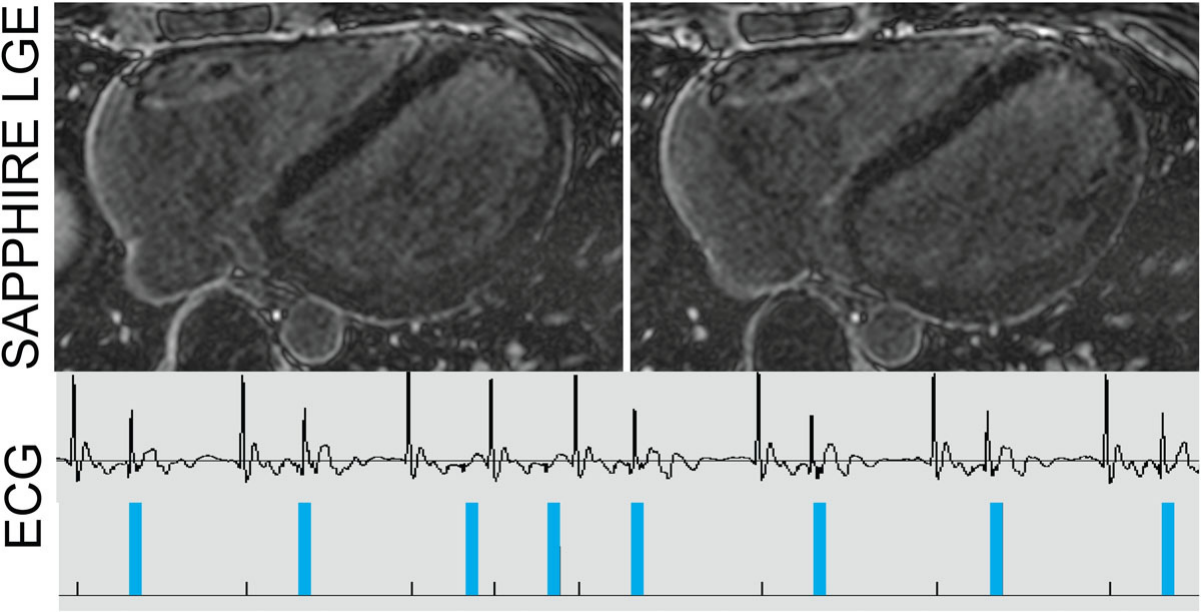
Images from a patient with arrhythmic ECG (lower row) acquired with SAPPHIRE LGE (upper row). The blue bars in the ECG indicate the time of k-space sampling. No ghosting artifacts can be seen in SAPPHIRE LGE images.

Figure 1a shows the results of the numerical simulations and Figure 1b the phantom measurements. The simulated arrhythmia consistently induces ghosting artifacts in conventional LGE. No artifacts are present in the SAPPHIRE LGE images. Figure 2 shows representative slices of a 3D SAPPHIRE LGE acquisition in a patient with heart rate variability and premature atrial beat.

## Conclusions

The proposed SAPPHIRE sequence removes the sensitivity of the conventional LGE sequence to heart rate variability and arrhythmia by erasing the magnetization history at the beginning of each heart-beat with the application of a saturation pulse.

## Funding

Deutsche Telekom Stiftung; NIH:R01EB008743-01A2; NIH: K99HL111410-01

